# Port Wine

**Published:** 1870-07

**Authors:** 


### PORT WINE.

As it is almost impossible to get pure wines in this country, it would be better to discard and resolutely refuse to use them, even medicinally.

In a recent examination of a quality of Port Wine sold in Stonington, Professor Silliman, of Yale College, found on a careful chemical analysis, that one gallon of it contained 21 per cent. of alcohol, 10 per cent. of molasses, and the remainder water, impregnated with oil of vitriol and the oxide of lead, of which there was about forty-five grains in a gallon. The liquor had more lead in it than most waters that are poisoned by it. Only home-made wines, those made in the family, are safe.

